# New Preparation Methods for Pore Formation on Polysulfone Membranes

**DOI:** 10.3390/membranes11040292

**Published:** 2021-04-18

**Authors:** Natalia Vainrot, Mingyuan Li, Arun M. Isloor, Moris S. Eisen

**Affiliations:** 1 Schulich Faculty of Chemistry, Technion-Israel Institute of Technology, Haifa 32000, Israel; natalyvain@gmail.com (N.V.); isloor@yahoo.com (A.M.I.); 2 Department of Chemistry, Guangdong Technion-Israel Institute of Technology, Shantou 515063, China; mingyuan.li@gtiit.edu.cn; 3 Membrane and Separation Technology Laboratory, Department of Chemistry, National Institute of Technology Karnataka, Surathkal, Mangalore 575 025, India

**Keywords:** polysulfone membranes, pore formation, chemical etching and hydrolysis, ultrafiltration and nanofiltration

## Abstract

This work described the preparation of membranes based on aromatic polysulfones through the phase-inversion method induced by a nonsolvent, generating the phase separation (NIPS) process. Three new techniques, including the nano iron acid etching method, base hydrolysis method of crosslinked polymers, and base hydrolysis method of a reactive component in a binary polymer blend, were developed for pore creation on membranes. The modified polymers and obtained membranes were carefully characterized. The uniform pores were successfully created by base hydrolysis of the crosslinked polymers and obtained at the size of the crosslinker. Moreover, homogeneous pores were created after base hydrolysis of the membranes prepared from binary polymer blends due to the internal changes in the polymer structure. The separation performance of membranes was tested with different inorganic salt solutions and compared with commercially known membranes. These new membranes exhibited high water flux (up to 3000 L/m^−2^·h^−1^ at 10 bar and at 25 °C) and reasonable rejections for monovalent (21–44%) and multivalent ions (18–60%), depending on the different etching of the hydrolysis times. The comparison of these membranes with commercial ones confirmed their good separation performance and high potential application for water treatment applications.

## 1. Introduction

With the milestone discovery by Loeb and Sourirajan in the 1960s, the invention of asymmetric membranes has made a great impact on the growth of membrane science and technology [1,2]. Asymmetric membranes exhibited excellent separation performances due to their unique structures consisting of a very thin, relatively dense skin layer supported by a more open porous sublayer [3] and thus have been extensively developed and widely applied both in academia and industry in the past decades [4].

Asymmetric membranes are mostly fabricated by a process named phase inversion, which can be achieved through three principal methods such as non-solvent-induced phase separation (NIPS) [5,6,7,8,9,10,11,12,13,14,15,16], evaporation-induced phase separation (EIPS) [17,18,19,20,21,22,23,24,25], and thermally induced phase separation (TIPS) [26,27,28,29,30,31,32,33,34]. In the NIPS technique, polymer homogeneous solutions are thermodynamically unstable because of many external factors, and polymer-lean and polymer-rich phase separation is operative [5]. The polymer-rich phase forms the matrix of the membrane, while the polymer-lean phase rich in solvents and nonsolvents fills the pores. When the casting solution is immersed into a nonsolvent coagulation bath, the interchange of solvents and nonsolvents, due to diffusion, causes the casting solution to go through a phase transition by which the membrane is formed [6,7,8]. Solvent–nonsolvent exchange occurs most rapidly at the interface, and the polymer precipitates much faster at the top surface than in the underlying substrate. This produces an asymmetric membrane with a dense surface layer on top of a microporous support. The dense skin layer determines the separation performance, while the porous sublayer provides mechanical support and influences the overall flow resistance. Membrane structure, especially dense layer thickness, sublayer morphology, pore size, and distribution, can be tailored for a specific application depending on the optimization of various polymers, solvents, nonsolvents, and preparation conditions [9,10,11,12,13,14,15,16]. In the EIPS process, most of the casting solutions consist of three or more components: polymer, volatile solvent, and less volatile nonsolvent as a pore former [17]. Compatibility of the homogeneous casting solution decreases as evaporation of the solvent proceeds, and inversion into a two-phase solution occurs due to the presence of nonsolvents and a strong polymer–polymer interaction force in the casting solution [18]. During the phase inversion, there is a loss of solvent, and the spherical-shaped micelles will attract each other, causing deformation and the diffusion transfer of molecules among the neighboring micelles, producing the expected intermingling at the interface of the polymer molecules. [19] The formation of a large number of such micelles having now a large surface area initiates the breaking of their walls, leading to the formation of gel-type networks. The TIPS method is a significant development in the technology of phase-inversion membranes, which applies to a wide range of polymers with poor solubility [26]. In essence, phase separation in the thermal process is evoked by utilizing a latent solvent and thermal energy. The latent solvent is a substance that acts as a solvent at elevated temperatures and a nonsolvent at lower temperatures. By removing heat, the loss of solvent power caused incompatibility of the system and resulted in phase inversion [27]. The nonvolatile latent solvents can be removed with ease from the final gel by extraction with a suitable liquid solvent.

However, all of the methods described above do not permit the effective control of the pore size or the pore size distribution on the obtained membranes. Great efforts have been made to develop new methods for homogeneous pore creation on polymeric membranes in the past decades [35,36]. Among them, some direct methods such as predesigned templates, chemical etching/hydrolysis, and polymer blends or copolymers are widely used to create pores on polymeric flat-sheet membranes due to their simple operation and economical process, and the resulting pore structures are also able to be tailored [37].

Membranes with highly ordered porous structures can be fabricated by the introduction and subsequent removal of ordered solid structures with accessible monodispersity. The nano- and micropillar arrays method was reported to provide membranes with cylindrical-shaped pores of uniform size, similar to the polymer membranes formed by track etching [38]. These ‘track-etched’ membranes still suffer, however, from relatively low porosity and thus low effluence. A simple method based on ‘reverse templating’ was also reported to provide membranes with regularly packed and uniformly sized cylindrical pores that occur for all the thickness of the membrane. Interestingly, it was possible to tune the pore diameter between 100 nm up to a few microns by varying the size of the templating sacrificial pillars [39]. As a proof of principle, a crude array of polysulfone pillars was formed to investigate the adaptability of the membrane process to other templates [40]. Additionally, the utilization of various nanoparticles such as NaCl [41], NaHCO_3_ [42], limestone [43], SrCO_3_ [44], and CaCO_3_ [44,45,46] as direct but random templates is also reported to be applicable in polysulfone or cellulose acetate. After the polymer matrix was stabilized, the removal of the solid salt particles by immersion in water or dilute acid generally led to the formation of closely controlled and interconnected pores.

The selective removal of one polymer component with a certain solvent from solidified polymer mixtures will provide separation between different polymer components with the formation of porous matrix polymer structures. Different porous systems can be formed depending on the mixing and interaction of the polymer blends [47]. Similarly, the removal of one of the blocks in a block-copolymer through thermal decomposition or chemical modification is also an efficient process toward the creation and structural control of pores within one specific polymeric membrane [36,48,49,50]. A typical example for homogeneous pore creation through crosslinked block copolymers was reported by Cavicchi and Lodge [48]. In this methodology, an ordered nanoporous material consisting of crosslinked polyisoprene (PI) was obtained from a macroscopically aligned poly(isoprene-*b*-dimethylsiloxane) (PI-PDMS) precursor by cross-linking the PI block and chemically degrading the PDMS block. The use of PDMS as a sacrificial block and TBAF as an etching reagent has been a novel and facile methodology for preparing nanoporous materials.

**Scheme 1:**
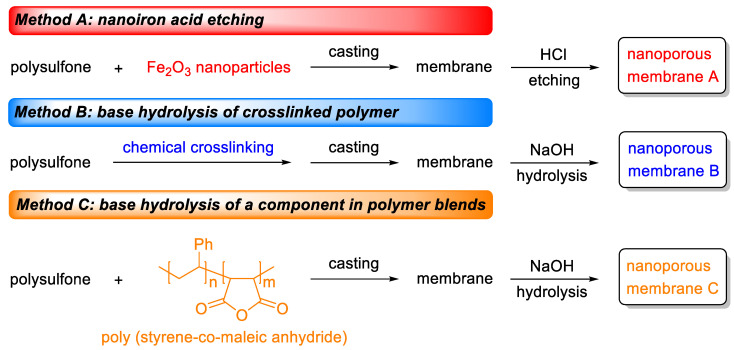
Three new methods for uniform pore formation on polysulfone membranes.

In this contribution, three new techniques for homogeneous pore creation on aromatic polysulfone membranes by the phase-inversion method were developed. As shown in Scheme 1, these routes include (a) the preparation of a polysulfone membrane under the presence of selected Fe_2_O_3_ nanoparticles and their dissolution by hydrochloric acid from polymer matrix (Method **A**); (b) The preparation of a polysulfone membrane through cross-linking between the acid groups of a carboxylated polysulfone with an alkylated diol. Partially cleavage of the cross-linkage motifs with a strong base induces the formation of numerous nanopores with uniform size (Method **B**); (c) The preparation of a polysulfone membrane through base hydrolysis of anhydride bonds inside the membrane composed from polysulfone and poly (styrene-co-maleic anhydride) blends (Method **C**). We wish to demonstrate that the nanopore membranes obtained by these methods are promising for water treatment applications with high water flux and reasonable salt rejections. 

## 2. Experimental Section

### 2.1. Materials

N-methyl-2-pyrrolidone (NMP) (99%) was purchased from Sigma-Aldrich (St. Louis, MO, USA) and fractionally distilled from barium oxide under reduced pressure (20 mmHg). Tetrahydrofuran (THF) (95%) anhydrous was purchased from Sigma-Aldrich and distilled under nitrogen from Na/K alloy. Pyridine (95%) was purchased from Fluka- Europe Amsterdam, Holland and distilled from barium oxide under nitrogen. *n*-Butyllithium was obtained commercially from Sigma-Aldrich as a 1.6 M solution in hexane and used as received. Thionyl chloride (95%) was purchased from Sigma-Aldrich and distilled under nitrogen. Ethylene glycol (99%) was purchased from Sigma-Aldrich, dried with MgSO_4_, and distilled under vacuum. Polysulfone polymer **1** (*M*_n_ = 20,000, PDI = 2.0) of analytical purity (95%) and polystyrene-co-maleic anhydride copolymer (*M*_n_ = 1600, PDI = 1.0) of analytical purity (95%) were purchased from Sigma-Aldrich and used as received. Dimethyl sulfoxide, DMSO (99%), hydrochloric acid (32%), sodium hydroxide solutions (99%), NaCl (99%), and CaCl_2_ (99%) salts of analytical purity were obtained from Sigma-Aldrich and used as received. 

### 2.2. Polymer Synthesis


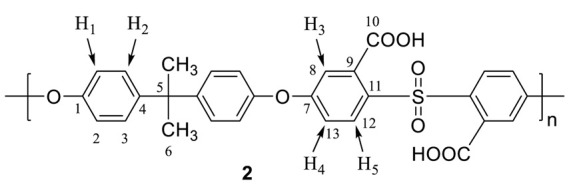


Polysulfone carboxylation (**2**). Dried polysulfone **1** was placed into a 100 mL three-necked Schlenk equipped with a dropping funnel, a thermometer, a N_2_ inlet, and a magnetic stirrer. A 2.0 g (4.52 mmol) amount of polysulfone **1** was dissolved in anhydrous THF (75 mL), and the temperature of the solution was reduced to −50 °C. *n*-Butyllithium (2.5 molar equivalent, 11.3 mmol, 7.06 mL of 1.6 M in hexane) diluted with 10 mL of THF was added dropwise over 12 min, during which time the mixture turned to a red-brown color. The polymer was quenched after 30 min by the slow addition (10.0 g) of CO_2(S)_ for 30 min and then warmed slowly to room temperature. The THF was evaporated on a Schlenk line to afford the white slurry. The polymer was precipitated into dilute aqueous HCl (10%) solution, washed with distilled water, and finally dried at 50 °C in a vacuum oven to obtain polymer **2** as a white solid (2.0 g, yield 98%). ^1^H NMR (500 MHz, DMSO) δ: 8.03 (d, ^3^*J* = 9 Hz, 2H, *H*_5_), 7.31 (d, ^3^*J* = 8 Hz, 4H, *H*_2_), 7.16 (dd, ^3^*J* = 3 Hz, ^5^*J* = 9 Hz, 2H, *H*_4_), 7.09 (d, ^4^*J* = 3 Hz, 2H, *H*_3_), 7.07 (d, ^3^*J* = 8 Hz, 4H, *H*_1_), 1.61 (s, 6H, C*H*_3_), 13.9 (br, 2H, O*H*) ppm. ^13^C NMR (125 MHz, DMSO) δ: 167.7 (*C*_10_), 161.3(*C*_11_), 152.4(*C*_4_), 147.4(*C*_1_), 136.8(*C*_9_), 133.5(*C*_12_), 132.5(*C*_7_), 128.9(*C*_3_), 120.1(*C*_2_), 118.2(*C*_13_), 116.9(*C*_8_), 42.3(*C*_5_), 30.8(*C*_6_) ppm. IR (KBr): 3536 (O–H str.), 1725 (C=O str, carbonyl group).


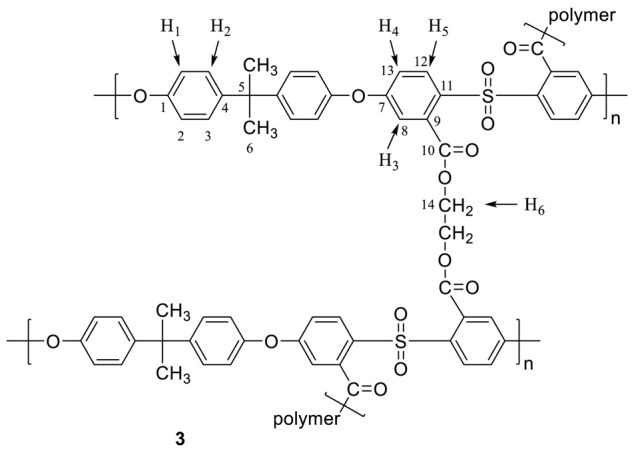


Polysulfone (**3**)**.** A 1.0 g (1.88 mmol) amount of polymer **2** was placed into a 50 mL Schlenk and dissolved in 30 mL of anhydrous THF. The Schlenk was connected to a trap with a NaOH solution (2.0 M) for the absorption of HCl and SO_2_. Then, 0.5 mL of anhydrous pyridine (6.2 mmol) and 0.3 mL of SOCl_2_ (4.1 mmol) were added dropwise into the Schlenk at room temperature. The temperature was slowly increased and maintained at 60 °C for 3 h. Excess of SOCl_2_ and THF were distilled off under vacuum at 50 °C for 30 min to obtain the resulting crude acid chloride polymer. Then, freshly distilled THF (30 mL) was added to dissolve the acylated polymer. After the 30 min needed for the complete dissolution of the polymer, a solution of 0.1 mL of ethylene glycol (1.8 mmol) in 10 mL of anhydrous THF was added dropwise to the polymer solution at room temperature under vigorous stirring and allowed to stir for an additional 24 h. The polymer was recovered by precipitating into distilled water, washing several times, and finally dried at 50 °C in a vacuum oven to obtain polymer **3** as a brown solid (1.0 g, yield 90%). ^1^H NMR (500 MHz, DMSO) δ: 7.88 (d, ^3^*J* = 9 Hz, 2H, *H*_5_), 7.04 (m, 12H, *H*_1_, *H*_2_, *H*_3_, *H*_4_), 4.4 (s, 4H, *H*_6_), 1.61 (s, 6H, C*H*_3_) ppm. ^13^C-(CP-MAS) NMR: 120 ppm (Δν_1/2_ = 1500 Hz) aromatic ring, 64 ppm (Δν_1/2_ = 375 Hz) ethylene group. IR (KBr): 2966-2926 (ethylene group, aliphatic C–H str.), 1737 (C=O str, ester group).

### 2.3. Apparatus

All flow tests were performed with a flat-pressure cell with an active area of 19.63 cm^2^, while the pressure applied was in the range of 2–15 atm. A conductometer (model DDS-11A) Mettler-Toledo was used for the measurement of conductivity of permeation and feed. NMR and ^13^C-(CP-MAS) NMR methods were used to analyze the structures of the synthesized polymers. Spectra were recorded on Bruker (Billerica, MA, USA) AV 300 and AV 500 NMR spectrometers. An infrared spectrometer (Bruker, Vector 22, Billerica, MA, USA) was used for functional group determination. AFM (Model Autoprobe CP) Park Scientifics, Santa Clara, CA, USA and HRSEM (Model Leo 982) Gemini-Zeiss, Hannover, Germany were used for the analysis of the topography of the surface layer of the membranes.

### 2.4. Measurements

After the membranes were compressed by air for 30 min, feed and permeate solutions were collected. The conductivity of these two solutions was measured by a conductometer. The water flux was calculated by measuring the volume of permeate that penetrated the membrane per unit of time. These experiments were conducted with identical feed solutions initially containing 0.2% NaCl and CaCl_2_. According to the conductivity–concentration dependence, the concentrations were obtained. Then, the rejection of salt *R* was calculated by using the following equation:

*R* = (*C*_f_*− C*_p_) / *C*_f_ × 100% (1)

where *C*_f_ and *C_p_* are the concentrations of the feed solution and permeate, respectively.

The membrane permeability to solution flow *L*_p_ was calculated by using the following equation:

*L*_p_ = *J*_v_ / (P − σ × Δπ) (2)

where *L*_p_ is the units of flux per unit pressure, such as L/(m^2^·h·bar); *J*_v_ (L/(m^2^·h)) is the flux through the membrane; P (bar) is the applied mechanical pressure; Δπ (bar) is the osmotic pressure; and σ is the reflection coefficient.

The osmotic pressure can be calculated by using the following equation:

Δπ = R × T × Cs × γ(3)

where R ((L·atm)/(mol·°K) is the ideal gas constant, T (°K) is the solution temperature, γ is the number of ions formed in the dissociation of one mole of salt, and *C*_s_ (M) is the total molar concentration of ions in solution.

After each run, the whole cell was rinsed thoroughly with demineralized water, and the membrane was washed to remove any deposition. The conductivity of water transferred through the membrane was measured to confirm the absence of adsorbed ions inside the membrane.

### 2.5. Nonaqueous Conductometric Titrations for Carboxylic Group Determination

Nonaqueous conductometric titrations were used to quantitatively determine the carboxylic acid group content in the polysulfone polymers by a back-titration method. The polymer containing these groups was first dissolved in DMSO solvent and then reacted with an excess of sodium hydroxide. An excess of sodium hydroxide was subsequently titrated with hydrochloric acid. A sharp end titration point was observed, confirming the strong acid–base reaction. The reactions for the back-titration are given in the following (Equations (4) and (5)):

(4) $$\mathrm{R-COOH}+\mathrm{NaOH}\to \mathrm{R-COONa}+H_{2}O+\mathrm{NaOH}$$

(5) $$\mathrm{NaOH}+\mathrm{HCl}\to \mathrm{NaCl}+H_{2}O$$

The number of the functional group determined by titrations was 2.00 functional groups per one repeating unit of the polymer.

## 3. Results and Discussions

### 3.1. Nanoiron Acid Etching Method

This method includes the synthesis of a polysulfone membrane in the presence of selected Fe_2_O_3_ nanoparticles (50 nm size), which were introduced into the polymer network. A casting solution was prepared to contain, by weight, 20% polysulfone, 2% Fe_2_O_3_ nanoparticles, and 78% N-methyl pyrrolidone (NMP) as the solvent. These nano iron particles were formed in dilute FeCl_3_ solution by the following reaction (Equation (6)):

(6) $$2\;{\mathrm{FeCl}}_{3}+3\;H_{2}O\to {\mathrm{Fe}}_{2}O_{3}+6\;\mathrm{HCl}$$

**Figure 1:**
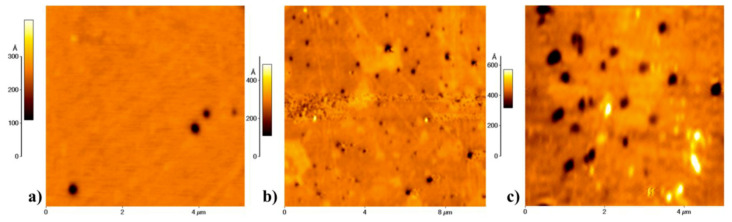
Atomic Force Microscope AFM pictures of the membrane obtained by the acid etching of nano iron particles at different times (pore size): (**a**) before etching (20–50 nm); (**b**) after 1 h of etching (20–100 nm); (**c**) after 6 h of etching (80–100 nm).

**Figure 2:**
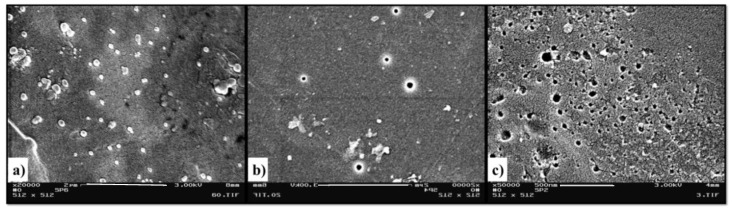
High Resolution Scanning Electron Microscopy HRSEM pictures of the membrane obtained by acid etching of nano iron particles at different times: (**a**) before etching; (**b**) after 60 min of etching; (**c**) after 2 h of etching. Bar scale: (**a**) 2 µm, (**b**) 2 µm, and (**c**) 500 nm

**Figure 3:**
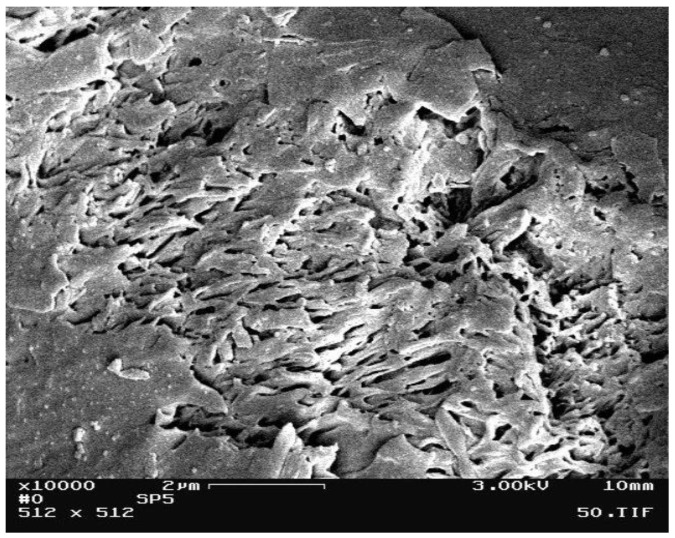
HRSEM picture of the cross-section of a membrane obtained by the acid etching of nano iron particles after 2 h of etching. The left side is the top of the membrane.

**Figure 4:**
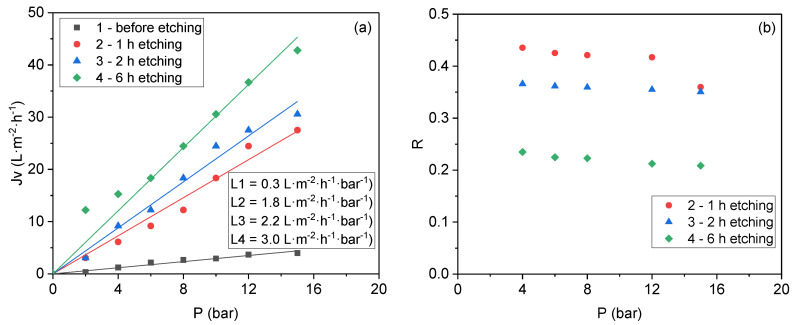
Plots of the flux (**a**) and 0.2% CaCl_2_ salt rejection (**b**) against pressure for membranes obtained at different acid etching times.

Fe_2_O_3_ nanoparticles with the desired size were obtained through control of the growth conditions [51]. Membrane samples were cast onto the glass surface to a thickness of 200 μm. The solvent was evaporated at 300 °C under nitrogen for 2 min before the cast film together with the glass plate was immersed in ice-cold water. Phase inversion started immediately, and the thin polymeric film was separated from the glass after a few minutes. It was repeatedly washed with demineralized water and wet stored. The actual thickness of the membranes was measured using a micrometer. The membranes were press-compacted by compressed air for 30 min to achieve the final structure. Dissolution of Fe_2_O_3_ nanoparticles with the etching agent HCl led to new pore creation, and corresponding AFM and HRSEM measurements are shown in Figure 1, Figure 2 and Figure 3, respectively. Flux and rejection measurements were performed at different acid etching times, and the results are shown in Figure 4.

As shown in Figure 1, the nano iron particle distribution inside of the polymer matrix is inhomogeneous. The nano iron particles formed aggregates or clusters, thus increasing their pore size after acid etching. The AFM and SEM pictures in Figure 1 and Figure 2 confirm the enlargement of pore size, with an increase in etching time due to nano iron aggregation into the big clusters. One important issue regards the cross-section of the membrane after the hydrolysis (Figure 3).

As can be observed, the hydrolysis of the nanometric iron allows forming continuous pores through the thickness of the membrane, indicating better fluxes as a function of etching time and the almost constant rejection of the salt, as expected, because no bigger holes are induced. It is important to address that the idea of pore sizes via the use of different sizes of salts is reaching an admirable stage [41,44,45,52,53]; however, the use of a magnetic iron entity will allow us, in the future, to control not just the size of the pore but the position of the pore in the membrane and the total geometrical distribution of the pores on the membrane.

The water flux increased almost linearly with pressure in the range of 0–15 bar; meanwhile, it also increased with the increase of etching time under the same operating pressure (Figure 4a). Under the pressure of 15 bar, the water flux was measured at the range of 27.5–42.8 L·m^−2^·h^−1^ depending on the acid etching time of the different membranes, which were all significantly higher than that of the membrane before etching (4.0 L·m^−2^·h^−1^). These results suggested an increase in pore size, decreasing skin layer resistance, and enhanced interconnectivity of pores by the increasing etching time.

The CaCl_2_ salt rejection of the membranes obtained by Method **A** was in the range of 20.8–43.5%, which was almost unaffected by pressure but markedly decreased with the increase in etching time (Figure 4b). Membranes with no functional groups on the polymer chain exhibited a rejection to salts and other dissolved substances by a sieving mechanism according to pore size distribution. Hence, the decrease in salt rejection after etching was mainly caused by the creation of numerous pores. An increase in the number of pores on the membrane and/or an increase in their pore size allows more solute molecules to pass through, thus lowering rejection. However, the salt rejection of this membrane was too high, although the pore sizes were as large as 100 nm. These salt rejection measurements suggested that the pores were not interconnected with each other across the membrane; thus, the closed pore structure in the membrane is maintained.

In a comparison of the separation performance between commercial membranes and the membranes prepared by Method **A**, which were all made from polysulfone by the phase-inversion method [54], this nano iron acid etching method helped to increase the permeability of solution through the membrane. The membranes prepared by Method **A** showed a considerably higher flux (30 vs. 6 L·m^−2^·h^−1^) and lower operating pressure (11 vs. 34 bar) than those of the commercial ones while keeping almost the same salt rejection (25 vs. 22%) as the latter (Entries 1 and 2, Table 1).

### 3.2. Base Hydrolysis Method of the Crosslinked Polymer

**Scheme 2:**
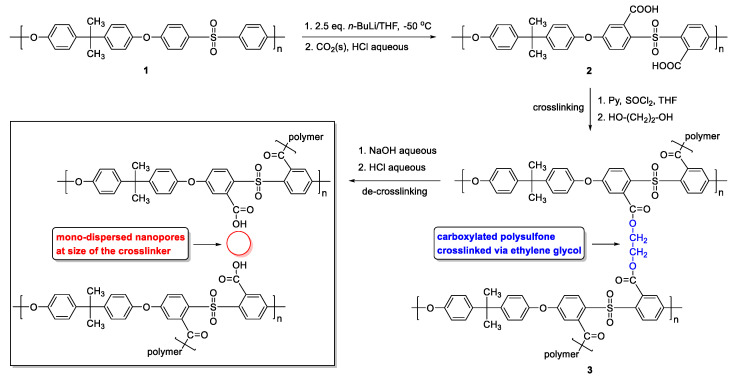
Preparation of monodispersed nanoporous membranes by base hydrolysis of crosslinked polysulfone carboxylated.

**Figure 5:**
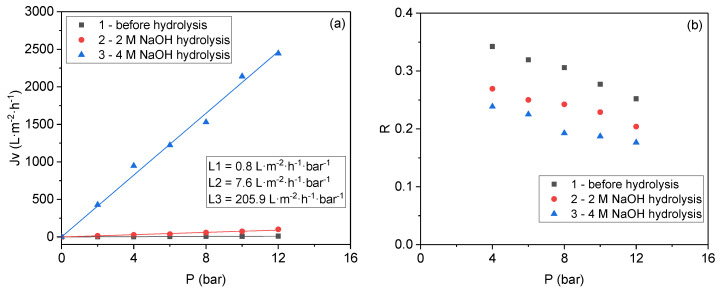
Plots of the flux (**a**) and 0.2% CaCl_2_ salt rejection (**b**) against pressure for membranes obtained by hydrolysis with different concentrations of aqueous NaOH.

In this developed methodology, membranes were prepared through the wet phase-inversion method, which included the preparation of a casting solution consisting of 20 wt.% of polysulfones with ethylene glycol and 80 wt.% of DMSO as the solvent. This solution was slightly swollen; therefore, 20 wt.% of a solution consisting of 20 wt.% of polysulfones and 80 wt.% of NMP was added to afford the homogeneous mixture for wet-casting. The homogeneous solution was cast onto the glass surface, followed by solvent evaporation at 300 °C for 2 min. The casted membrane was immersed into the distilled water bath at 0 °C overnight. The membrane was press-compacted by compressed air for 30 min to achieve the final structure. Base hydrolysis of ethylene glycol ester bonds with different concentrations of aqueous NaOH was performed to achieve monodispersed nanopores at the size of the crosslinker (ethylene glycol) (Scheme 2). Subsequently, the carboxylated polymer membranes were converted into their acid form by a simple acidification procedure, entailing the immersion of membranes for 30 min in hydrochloric acid followed by soaking for 12 h in deionized water. Water flux and salt rejection measurements were performed before and after NaOH hydrolysis, and the results are shown in Figure 5.

An increase in flux caused by an increase in porosity after a continuous base hydrolysis time was observed in our preliminary experiments, and the suitable hydrolysis time was 6 h. The water flux increased linearly with pressure and increased with the concentrations of the aqueous NaOH under the same pressure (Figure 5a). By hydrolysis with 4 M NaOH aqueous solution, the water flux of the obtained membranes was measured up to 2445.2 L·m^−2^·h^−1^ under a pressure of 12 bar. This value is much higher than those of the membranes obtained by hydrolysis with 2 M NaOH solutions and the membranes before base hydrolysis, which gave flux values of 100.9 and 10.7 L·m^−2^·h^−1^, respectively. These results indicate that the hydrolysis of crosslinked structures of the polymer and the formation of new pores in the membrane was successfully designed.

Although the permeability of the membrane increased considerably after hydrolysis with a 4 M NaOH solution, the difference of the salt rejection between the membranes obtained by hydrolysis with 4 and 2 M NaOH is negligible (Figure 5b). The salt rejection values maintained almost constant at around 20%, indicating that homogeneous pore size was created from the hydrolysis of the ethylene glycol ester bonds. In most state-of-the-art nanofiltration (NF) membranes, negative groups are primarily from carboxylic acid groups, which are readily complex with calcium and sodium cations. The appearance of carboxylic acid groups after base hydrolysis and the acidification process in Method **B** is also the reason to keep the rejection high. This result is in agreement with our previous results, which represented the membrane only consisting of polysulfone with carboxylic acid groups. 

In comparison to commercial polysulfone membranes containing hydrophilic sulfonic groups, the membranes obtained by Method **B** exhibited higher water flux (2200 vs. 500 L·m^−2^·h^−1^) and higher salt rejection (20 vs. 15%), respectively (Entries 3 and 5, Table 1). Therefore, base hydrolysis of the crosslinked polymer method described above is an alternative and highly valuable method to afford the membrane with improved separation performances by homogeneous pore creation.

**Figure 6:**
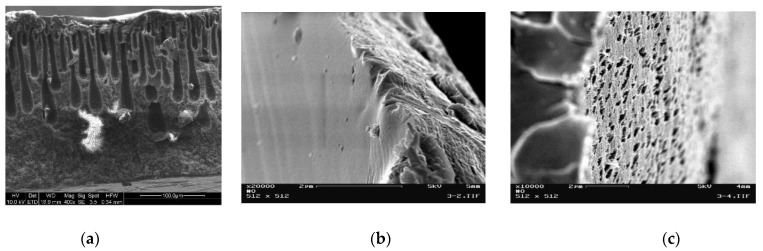
HRSEM images of the cross-section of the membranes produced by Method **B** using bis-carboxylated polysulfones: (**a**) before the crosslinking; (**b**) after crosslinking with a diol. The top of the membrane is on the left side (**c**) after 6 h of hydrolysis of some of the ester bonds. The top of the membrane is on the right side.

Extremely important is to investigate the cross-section of the polysulfone carboxylated before the cross-linking, after the cross-linking, and after the hydrolysis. (Figure 6). The HRSEM of the polysulfone biscarboxylated shows the regular finger and a very narrow active membrane as expected for the phase-inversion formation of those membranes (Figure 6a). After the cross-linking process, we obtained a closed membrane with almost no porosity (Figure 6b); however, after 6 h of hydrolysis, the formation of nanopores was perceived, corroborating our flux and rejection results (Figure 6c).

It is important to point out that the flux/rejection in these membranes is very interesting. The flux of the membranes is in the range of nanofiltration (Membrane **A**) to ultrafiltration (Membrane **B**). Hence, based on the flux alone, we should have expected a much higher rejection for the nanofiltration-behaving membranes and no rejection for the ultrafiltration membrane. However, in all cases, a rejection of about 20% for a 0.2% solution of CaCl_2_ is observed. Based on pore size, it is clear that the formed pores of all the membranes are much larger, so no rejection should have been expected. Hence, it seems that the active layer that is formed upon the formation of the membrane via the inverse technique is mainly responsible for the rejection of the salts. 

### 3.3. Base Hydrolysis Method of a Component in Polymer Blends

**Scheme 3:**
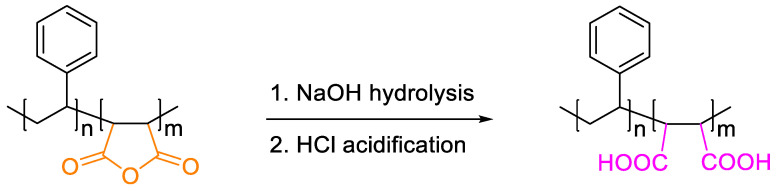
Base hydrolysis and acidification of anhydride bonds in poly(styrene-co-maleic anhydride).

In this method, membranes were prepared from blends of two different polymers, polysulfone and polystyrene-*co*-maleic anhydride, at an 80/20 percent ratio, and the influence of base hydrolysis of the anhydride bonds on their separation performance was investigated. We expected that the introduction of the COOH groups and the changes in polymer structure would increase their hydrophilicity induced through the hydrogen bonding and improve the salt rejection caused by the negative charges of dissociated carboxylic groups (Scheme 3).

**Figure 7:**
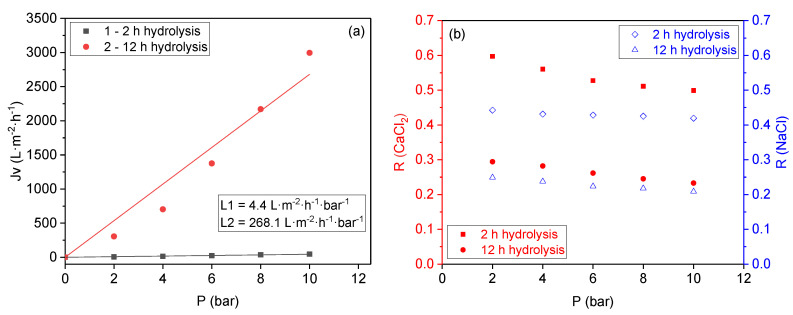
Plots of the flux (**a**) and 0.2% CaCl_2_ and 0.2% NaCl salts rejection (**b**) against pressure for membranes obtained at different hydrolysis times.

The membranes were prepared through the wet phase-inversion method by the preparation of casting dope consisting of 20 wt.% of polymer blends (20 wt.% of poly(styrene-*co*-maleic anhydride) and 80 wt.% of polysulfones) using 80 wt.% of NMP as the solvent. The homogeneous solution was cast using a casting knife, followed by solvent evaporation at 300 °C for 2 min. The membranes were immersed in a distilled water bath at 0 °C overnight. Press compaction by compressed air was applied to achieve the final equilibrium structure. Base hydrolysis of the anhydride bonds by 2.0 M NaOH at different periods was performed to afford carboxylic acid sodium salt. The carboxylated polymer membranes were converted into their acid via an acidification procedure, entailing the immersion of membranes for 30 min in hydrochloric acid followed by soaking for 12 h in deionized water. The water flux and salt rejection measurements were performed at different hydrolysis times, and the results are shown in Figure 7.

The water flux increased with the increase of base hydrolysis time for both of the two kinds of a membrane (Figure 7a). After 2 h of base hydrolysis, a high-performance membrane with high selectivity in compensation for low permeability was obtained, which exhibited a water flux value of 45.8 L·m^−2^·h^−1^ under the pressure of 10 bar. After 12 base hydrolysis, however, the obtained membrane gave a much higher water flux value of 2995.4 L·m^−2^·h^−1^ at the same conditions. The increase in hydrolysis time increases the water flux through the membrane, indicating the creation of new pores in the membrane. 

With the base hydrolysis time increasing from 2 to 12 h, the CaCl_2_ salt rejection decreased from ~53% to ~26%, and the NaCl salt rejection decreased from ~43% to ~23%, respectively (Figure 7b). The same membrane showed relatively higher rejection to divalent ions as compared to the monovalent ions after 2 h of base hydrolysis. However, the rejection difference became negligible between divalent and monovalent ions after 12 h of base hydrolysis. Although the membrane rejection decreased by almost half of their original value with increasing hydrolysis time, the appearance of carboxylic groups after base hydrolysis helped to keep moderate rejection values.

**Figure 8:**
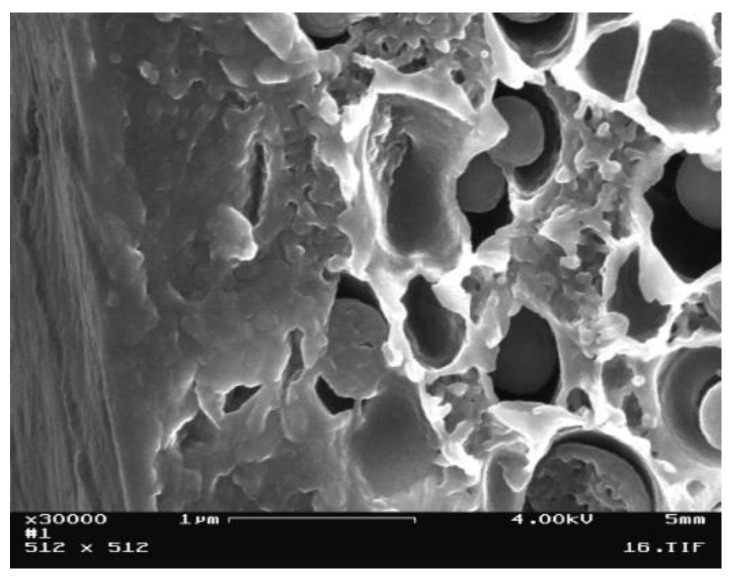
SEM picture of the cross-section of the membrane after hydrolysis with NaOH after 12 h. The top of the membrane is on the left side.

The cross-section of the membrane after hydrolysis shows the formation of a high-porosity membrane with 400–500 nm sized pores all through the thickness of the membrane (Figure 8).

The mechanism of pore formation by this method is still under investigation, and it is assumed that the pores were created by the structural changing of the polymer matrix in space, thus enabling the different polymer chains rearrangement after base hydrolysis. The pores were possibly formed by the new distances between the polymer chains. 

## 4. Conclusions

In summary, three different preparation methods toward pore creation on polysulfone-based membranes were developed, and the obtained nanopores membranes presented good separation performances and a high potential for water treatment applications. A comparison table on the performances of the membranes can be observed in Table 2.

The nano iron etching method presents a simple way to tailor the pore size. The distribution of the pores can potentially be tailored with magnetic fields. Notably, homogeneity in pore size distribution can be significantly improved by the base hydrolysis method of crosslinked polymer units, which afforded homogeneous pore creation on the membranes at the size of the crosslinker. The membrane prepared through the base hydrolysis method of anhydride bonds from polymer blends showed a slightly high salt rejection with a considerably higher flux, which was caused by changes in the polymer structure after base hydrolysis. The properties of prepared membranes were strongly dependent on etching and hydrolysis times, allowing the application of these membranes following specific purposes. In comparison with commercial membranes, the membranes prepared in this work exhibit wider operating pressure ranges and higher water fluxes while keeping alike salt rejection as commercial membranes. The salt rejection mechanisms are being studied for the different membranes and will be presented in a different study.

## Figures and Tables

**Table 1:** Comparison between laboratory prepared membranes and commercial membranes.

Entry	Membrane Type	Pressure (Bar)	Flux (L·m^−2^·h^−1^)	Rejection (%)
1 ^a^	Commercial polysulfone membrane	34	6	25
2	Polysulfone membrane by Method **A**	10	30	22
3 ^b^	Commercial polysulfone sulfonated membrane	10	500	15
4 ^c^	Patented polysulfone carboxylated membrane	11	2100	20
5	Polysulfone carboxylated membrane by Method **B**	10	2200	20

^a^ Kalle company; ^b^ Nitto Denko company; ^c^ Data from the literature [54].

**Table 2:** Comparison in the performance of the membranes obtained by the three disclosed methods.

Entry	Membrane Type	Pressure (Bar)	Flux (L·m^−2^·h^−1^)	Rejection 0.2% CaCl_2_(%)
1	Polysulfone membrane by Method **A**	10	30	43
2	Polysulfone carboxylated membrane by Method **B**	10	2200	20
3	poly(styrene-*co*-maleic anhydride) (12 h of hydrolysis) by Method **C**	10	2680	25

## Data Availability

Not applicable.
